# Editorial: Insights in integrative physiology: 2022

**DOI:** 10.3389/fphys.2023.1220684

**Published:** 2023-05-23

**Authors:** James T. Pearson, Jacqueline K. Phillips, Geoffrey A. Head

**Affiliations:** ^1^ National Cerebral and Cardiovascular Center, Suita, Japan; ^2^ Department of Physiology, Victoria Heart Institute and Monash Biomedicine Discovery Institute, Monash University, Clayton, VIC, Australia; ^3^ Macquarie Medical School, Macquarie University, Sydney, NSW, Australia; ^4^ Baker Heart and Diabetes Institute, Melbourne, VIC, Australia

**Keywords:** immune, cardiovascular, autonomic, respiratory, neural, integrative physiology, musculoskeletal

The second edition of the Frontiers Series: *Insights in Integrative Physiology (2022)* aims to continue with the theme of providing a comprehensive overview of recent developments and challenges in Physiology. This series of publications covers the interrelationship between the immune, neural, cardiovascular, respiratory, and musculoskeletal systems, highlighting both novel advances and revisiting previously established dogma. Through this commentary, we hope to encourage researchers to pursue innovative approaches to address the most pressing issues in the field of Integrative Physiology.

Our first article lies in the field of immunology, which intersects with all aspects of human physiology and pathological processes. The hypothesis article by Bajgar et al. raises the novel idea that the ancient specialisation of macrophages evolved during the transition from single to multicell organisms. The authors suggest that during this transition, macrophages diversified and expanded their role to immune protection, development, tissue and metabolic maintenance, organogenesis as well as many organ specific roles. Thus today, macrophages exhibit a high degree of functional versatility being able to recognise and eliminate pathogens, remove senescent and worn-out cells, participate in wound healing, and promote metabolic homeostasis. Importantly, populations of tissue-specific macrophages exhibit highly specialised organ functions and are therefore an essential part of higher order animal homeostasis. It is also clear that in some cases, macrophages, unexplainedly, cease their protective function and cause pathology. The authors surmise that many of the features of proinflammatory macrophages can be attributed to their unicellular origins, with activation of ancient vestigial functions that appear counterintuitive in the specific context a complex animal or human body. The authors suggest that the entire repertoire of macrophage functions evolved by repurposing and diversification of normal functions that evolved in amoebocytes early in evolution under conditions quite distinct from tissues of advanced multicellular organisms. They suggest that we may be able to use this perspective to find explanations for the behaviour of macrophages in many pathologies.

Atherosclerosis is a major contributor to the burden of cardiovascular disease and the leading cause of ischaemia. Considering alternative approaches to the treatment of atherosclerosis, the opinion piece by Shakeel and Corridon discuss current progress in overcoming major challenges for the widespread clinical adoption of vascular tissue engineering, regeneration techniques, vascular access lines, and vessel constructs. They share their insights on the most promising studies addressing these limitations in ischaemia therapies. The ideal vascular graft, they suggest, should possess a number of essential qualities including bio-inertness, cost-effectiveness, and mechanical robustness. As researchers develop advanced computational models to create reliable vascular grafts, the authors emphasise the importance of understanding the synergistic effects of graft design, cellular cascades, and mechanical and immunological responses in order to promote effective healing and functional remodelling.

Acute respiratory distress syndrome (ARDS) is a major cause of morbidity and mortality in critically ill patients, and mechanical ventilation can contribute to ventilator-induced lung injury (VILI). The study by Mlček et al. investigated the effects of a sequential lateral positioning strategy (literally changing position from side to side) on ventilation, perfusion distributions, and regional lung volumes in an early ARDS porcine model, examining the hypothesis that when combined with real-time personalisation of positive end-expiratory pressure (PEEP), sequential lateral positioning would reduce lung collapse and improve ventilation. Their physiology-based, personalised and effectively simple sequential lateral positioning strategy demonstrated a significant reduction in lung collapse in the model. This promising approach has the potential to decrease major mechanisms of VILI with future studies set to determine the optimal duration of each step in the strategy and to explore the most suitable ventilator settings.

The next three studies deal with the autonomic neural regulation and control of specific physiological processes. Continuing first with consideration of respiratory function, Zhao et al. sought to unravel the role of group II and III metabotropic glutamate receptors (mGluRs) within the carotid body, given both the fundamental role that glutamate plays as a central neurotransmitter and the key role that chemosensors are thought to contribute to cardiorespiratory syndromes including obstructive sleep apnoea and heart failure as a result of dysregulation in carotid body neurotransmitter release. Their work demonstrated for the first time in a rodent model that mGluRs that play an important inhibitory role on carotid body nerve activity in reflex responses to acute hypoxia. They further demonstrated the expression of mGluRs in human carotid body. The role of these receptor subtypes within the carotid body in disease states is a rich area for investigation, potentially leading to the development of targeted therapies that modulate the activity of these receptors to manage conditions related to hypoxia and impaired chemoreflex responses such as obstructive sleep apnoea, hypertension, and congestive heart failure.

Further examining the response to hypoxia, but in the form of intermittent bouts of hypoxia, Ostrowski et al., set out to determine if the nucleus tractus solitarii (NTS) is required for the development and maintenance of phrenic and sympathetic long-term facilitation after acute intermittent hypoxia (AIH). The NTS is a major medullary cardiovascular integrative site being the primary point of termination of baroreceptor and chemoreceptor afferents input. It has now been well established that repeated exposure to acute intermittent bouts of hypoxia induces long term facilitation (LTF) in phrenic and sympathetic nerve activity under basal conditions and enhances respiratory and sympathetic responses to hypoxia. However, the mechanism is not well understood. To test whether NTS itself is key to the LTP, neuronal activity in the NTS was inhibited with a GABAA receptor agonist muscimol both before AIH exposure or after development of AIH-induced LTF in isoflurane anaesthetised rats. Indeed, inhibiting the NTS before AIH prevented the development of LTF while inhibition of the NTS after the development of AIH induced LTF largely reversed but did not eliminate the LTF. The authors concluded that the NTS is indeed critical for the initiation of LTF and required for the full expression of the facilitation.

The third paper examining neural control is a controversial topic that has been the subject of varying perspectives. Kulkarni et al. delve into the functional anatomy and neurotransmitter mechanisms of the rostral ventrolateral medulla (RVLM). A part of many of the same pathways activated by the NTS, the RVLM is a key player in regulating blood pressure via tonic sympathetic drive to most organs, however, how the RVLM regulates sympathetic nerve activity (SNA) to different organs is not fully understood. The aim of the study was to determine the functional significance of ipsilateral *versus* contralateral projections from the RVLM, comparing splanchnic *versus* adrenal SNA, and whether GABA withdrawal mediated increased activity of functionally different sympathetic nerves during hypotension. The findings were that activation of ipsilateral RVLM leads to greater increases in adrenal SNA compared to activation of the contralateral RVLM, but activation of either side of the RVLM produced equivalent increases in splanchnic SNA. Blocking GABAA receptors prevented changes in splanchnic SNA during hypotension but had no effect on increases in adrenal SNA. The authors conclude that the RVLM regulates adrenal SNA and splanchnic SNA differently through at least two distinct mechanisms, challenging the previous idea of an all-or-none concept of regulation. With recent advances in spinal cord stimulation therapies, the study underscores the importance of understanding the differences in projection patterns and sympathetic nerve plasticity if we are to further develop these therapeutic approaches.

The last study we review is an examination of the use of surface electromyography (sEMG). sEMG records from the surface of the muscles and is therefore a composite signal from several different motor unit action potentials. It can be used to estimate muscle force and to date various models have been used in these calculations including polynomial fitting models, fast orthogonal search, and parallel cascade identification, however, these approaches are prone to error and new models are required. The article by Shirzadi et al. proposes a new real-time convex and interpretable model for estimating force estimation from sEMG, using the upper limb during isometric voluntary flexions and lower limbs during standing tasks. The new method was compared to eleven state-of-the-art methods, including linear-in-the-parameter models, Artificial Neural Networks and Supported Vector Machines, and nonlinear models. Importantly, the new method was not significantly different from the recorded force signal which was not the case for the other tested models and indeed, the proposed method outperformed the other methods. These findings have major and important implications for application in load sharing, robotics, rehabilitation, and prosthetics.

The common theme across these articles is the exploration of integrative physiology systems, investigating their functional importance and the underlying mechanisms that regulate them. Review papers considered the evolutionary origin of macrophages and their functional versatility alongside the challenges in developing bioartificial vascular grafts. Novel data was presented on the role of the RVLM and NTS in controlling SNA and respiratory activity and the involvement of mGluRs in the carotid body’s response to hypoxia. Two papers provided a highly translational perspective, examining the use of sEMG to estimate muscle force, and the effects of sequential lateral positioning on ventilation and perfusion distributions in ARDS. Collectively, these articles highlight the significance of understanding fundamental physiological mechanisms if we are to develop novel treatments, enhance rehabilitation, and explain the behaviour of biological systems in human pathologies.

